# The TRACTISS Protocol: a randomised double blind placebo controlled clinical TRial of Anti-B-Cell Therapy In patients with primary Sjögren’s Syndrome

**DOI:** 10.1186/1471-2474-15-21

**Published:** 2014-01-17

**Authors:** Sarah Brown, Nuria Navarro Coy, Costantino Pitzalis, Paul Emery, Sue Pavitt, Janine Gray, Claire Hulme, Frances Hall, Robert Busch, Pete Smith, Luke Dawson, Michele Bombardieri, Ng Wan-fai, Colin Pease, Elizabeth Price, Nurhan Sutcliffe, Clodagh Woods, Sharon Ruddock, Colin Everett, Catherine Reynolds, Emma Skinner, Ana Poveda-Gallego, John Rout, Iain Macleod, Saaeha Rauz, Simon Bowman

**Affiliations:** 1 Clinical Trials Research Unit, Leeds Institute of Clinical Trials Research, University of Leeds, Leeds LS2 9JT, UK; 2 Leeds Institute of Rheumatic and Musculoskeletal Medicine, University of Leeds, Chapel Allerton Hospital, Chapeltown Road, Leeds LS7 4SA, UK; 3 Centre for Experimental Medicine and Rheumatology, William Harvey Research Institute, Barts & The London, Queen Mary University of London, 2nd Floor John Vane Science Centre, Charterhouse Square, London EC1M 6BQ, UK; 4 Department of Clinical Medicine, University of Cambridge, Addenbrookes Hospital, Hills Road, Cambridge CB2 2QQ, UK; 5 School of Dental Sciences, University of Liverpool, Liverpool L69 3GN, UK; 6 NIHR Leeds Musculoskeletal Biomedical Research Unit, Leeds Teaching Hospitals NHS Trust, Leeds, UK; 7 Academic Unit of Health Economics, Leeds Institute of Health Sciences, University of Leeds, Leeds LS2 9LJ, UK; 8 Centre for Health Sciences Research, Leeds Institute of Health Sciences, University of Leeds, Leeds LS2 9LJ, UK; 9 Musculoskeletal Research Group, University of Newcastle, 4th Floor Catherine Cookson Building, Framlington Place, Newcastle-upon-Tyne NE2 4HH, UK; 10 Chapel Allerton Hospital, Chapeltown Road, Leeds LS7 4SA, UK; 11 Great Western Hospital, Marlborough Road, Swindon SN3 6BB, UK; 12 Royal London Hospital (Mile End), Bancroft Road, London E1 4DG, UK; 13 TRACTISS PPI representative, c/o Chapel Allerton Hospital, Chapeltown Road, Leeds LS7 4SA, UK; 14 Birmingham Dental Hospital, St Chad’s Queensway, Birmingham B4 6NN, UK; 15 Newcastle Dental Hospital & School, Richardson road, Newcastle upon Tyne NE2 4AZ, UK; 16 Academic Unit of Ophthalmology, Centre for Translational Inflammation Research, University of Birmingham, Birmingham B15 2TT, UK; 17 Department of Life Sciences, University of Roehampton, Whitelands College, Holybourne Ave, London SW15 4JD, UK; 18 Arthritis Research UK Primary Care Centre, Primary Care Services, University of Keele, Staffordshire ST5 5BG, UK; 19 New Queen Elizabeth Hospital, Mindelsohn Way, Edgbaston, Birmingham B15 2WB, UK

**Keywords:** Sjögren’s syndrome, Rituximab, Anti-B-cell, Double-blind, Placebo, Trial

## Abstract

**Background:**

Primary Sjögren’s Syndrome (PSS) mainly affects women (9:1 female:male ratio) and is one of the commonest autoimmune diseases with a prevalence of 0.1 – 0.6% of adult women. For patients with PSS there is currently no effective therapy that can alter the progression of the disease. The aim of the TRACTISS study is to establish whether in patients with PSS, treatment with rituximab improves clinical outcomes.

**Methods/design:**

TRACTISS is a UK multi-centre, double-blind, randomised, controlled, parallel group trial of 110 patients with PSS. Patients will be randomised on a 1:1 basis to receive two courses of either rituximab or placebo infusion in addition to standard therapy, and will be followed up for up to 48 weeks. The primary objective is to assess the extent to which rituximab improves symptoms of fatigue and oral dryness. Secondary outcomes include ocular dryness, salivary flow rates, lacrimal flow, patient quality of life, measures of disease damage and disease activity, serological and peripheral blood biomarkers, and glandular histology and composition.

**Discussion:**

The TRACTISS trial will provide direct evidence as to whether rituximab in patients with PSS leads to an improvement in patient symptoms and a reduction in disease damage and activity.

**Trial registration:**

UKCRN Portfolio ID: 9809 ISRCTN65360827.

## Background

Primary Sjögren’s Syndrome (PSS) mainly affects women (9:1 female:male ratio) and is one of the commonest autoimmune diseases with a prevalence of 0.1 – 0.6% of adult women in community studies using the American-European Consensus Group (AECG) criteria [1-3]. PSS is characterised by a combination of features including oral and ocular dryness, which can be disabling symptoms, ocular signs i.e. objective evidence for ocular involvement, abnormal appearance of salivary glands, salivary gland involvement and presence of antibodies to Ro and/or La. PSS patients may also experience severe, variable & unpredictable fatigue, which is similar in character and severity to that of patients with Systemic Lupus Erythematosus (SLE) [4]. Similarly, fibromyalgia (widespread chronic pain, unrefreshing sleep and 11 out of 18 tender trigger points) is found in 5% of PSS patients, again comparable to SLE [4]. Organ-specific systemic involvement is observed in 5-20% of patients. This includes rashes, peripheral neuropathy, non-erosive arthritis, interstitial cystitis, lung and renal disease. These patients almost always have evidence of B-cell hyper-reactivity with anti-Ro/La antibodies & hypergammaglobulinaemia.

For patients with PSS there is currently no effective therapy that can alter the progress of the disease. Symptomatic therapies for dry eyes, such as artificial tears, are reasonably effective. By contrast, therapies for dry mouth (sprays, gels or lozenges/pastilles) are poorly effective for most people. There is no effective therapy for fatigue.

Hydroxychloroquine and/or low dose prednisolone are often used in mild disease. At the severe end e.g. progressive neuropathy, IV methylprednisolone, cyclophosphamide, azathioprine, ciclosporine, mycophenolate or chlorambucil may be used.

Rituximab (MabThera®/Rituxan®) is a chimeric mouse/human monoclonal antibody against human CD20, a non-glycosylated transmembrane phosphoprotein, expressed on pre-B and mature B-lymphocytes. Rituximab depletes B cells by several potential mechanisms, including complement-mediated lysis, antibody-dependent cellular cytotoxicity (ADCC)-mediated killing, and apoptosis. Treatment with rituximab induces a rapid and sustained depletion of B cells. Median peripheral B-cell counts decline below normal following completion of the first dose, with recovery beginning after 6 months. B-cell levels return to normal between 9 and 12 months following completion of therapy.

Rituximab is currently approved for the treatment of relapsed or refractory non-Hodgkin’s lymphoma (NHL), chronic lymphocytic leukemia (CLL), and in combination with methotrexate (MTX) for the treatment of Rheumatoid Arthritis (RA) patients who inadequately respond to one or more anti-tumor necrosis factor (anti-TNF) therapies.

There is supportive evidence for the beneficial effects of rituximab in treating PSS patients from several small studies [5-10]. There is also early data that in patients with high pre-treatment levels of B-cell Activating Factor/B Lymphocyte Stimulator (BAFF/BLyS), B-cell recovery occurs sooner [11] following rituximab therapy and also that BAFF/BLyS levels increase following B-cell depletion [12]. A multicentre parallel-group randomised double-blind placebo controlled study of rituximab in 120 patients with PSS in France has completed [13]. In this study patients were randomly allocated to receive one course of rituximab or placebo infusions at weeks 0 and 2, with follow-up at 24 weeks. The primary outcome was a 30 mm improvement in two out of four visual analogue scales (VAS – Range 0-100 mm) (patient global assessment of disease activity, joint pain, fatigue and dryness) at 24 weeks. Preliminary results of the trial reveal that it failed to meet its primary endpoint over the 24 week period, potentially suggesting that the TRACTISS trial is justified in assessing the efficacy of two courses of treatment, each with two doses, spaced two weeks apart which is in contrast to the French trial which administered only one course of two doses. Furthermore, the TRACTISS trial is intended to be closely aligned to the design of the French study to permit a meta-analysis. As a consequence the eligibility criteria and potential impact on patient recruitment was modelled from data in the United Kingdom Primary Sjögren's Syndrome Registry (UKPSSR) [14] to ensure a robust recruitment strategy could be employed.

T-cell phenotypes and transcriptional profiles have been reported to be abnormal in the peripheral blood and affected tissues of patients with PSS, where T cells comprise the majority of infiltrating lymphocytes. B cells and T cells interact in lymphoid organs and within the inflamed tissue, suggesting that any clinical effects of B-cell depletion may be due to indirect effects on T cells. In a subset of patients in TRACTISS, T cells will be isolated from peripheral blood and their transcriptional profiles compared to healthy and disease controls to explore whether abnormal T-cell transcriptional activity at baseline (a) normalises following Rituximab therapy; and (b) predicts the patients’ subsequent clinical course. The results will also be compared with transcriptional activity of lesional T cells in an attempt to find peripheral blood correlates of T cell-mediated damage to lesional tissue.

PSS imposes a cost burden on patients and the health service. Annual direct healthcare costs of PSS have been estimated at £2,188 per annum per patient, comparable to a cost of £2,693 per annum per patient for those with RA [15]. Although this study predated the widespread use of biologic therapy in RA it suggests that treating PSS currently costs 50-80% of the total cost of treating an RA patient and further supports the observation that untreated PSS has a significant health economic impact.

## Methods/design

### Trial aims and objectives

The main aim of the study is to determine whether use of rituximab is of benefit to patients with PSS.

### Primary objective

To assess whether rituximab, compared with placebo infusion, improves symptoms of fatigue and oral dryness measured by visual analogue scales (VAS) in patients with PSS.

### Secondary objectives

To assess whether rituximab, compared with placebo:

• Increases salivary and lacrimal flow.

• Produces improvement in serological and peripheral blood inflammatory features.

• Improves quality of life and disease activity indices.

• Produces improvement in systemic features.

• Is cost effective.

To further validate questionnaires measuring disease activity and patient symptoms currently in development for use in the assessment of Sjögren’s Syndrome.

### Sub-studies objectives

To conduct a central analysis of local and systemic T-cell abnormalities in patients with PSS and their response to B-cell depletion:

• To compare peripheral blood T-cell transcriptional signatures in PSS patients with healthy and disease controls.

• To compare peripheral blood T-cell transcriptional signatures in PSS patients’ pre- and post- B-cell depletion.

• To characterise tissue distribution, phenotype, and gene expression of lesional T-cells in PSS patients pre- and post-B-cell depletion.

#### Optional labial gland biopsy and imaging

• To conduct a central review to assess whether rituximab has an effect on glandular histology and to determine whether ultrasound imaging is an effective tool in determining treatment response.

### Trial design

**Figure 1:**
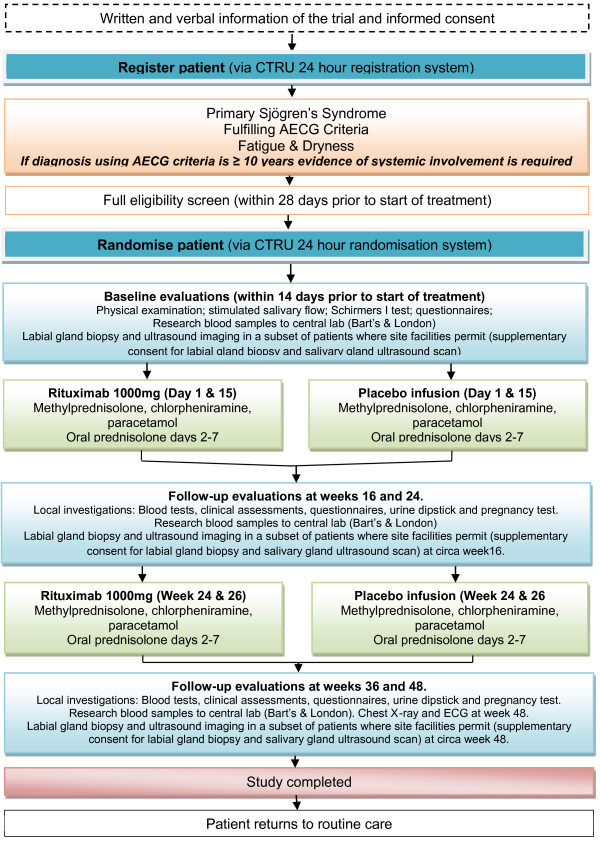
TRACTISS trial flow diagram.

The TRACTISS trial is a multi-centre, double-blind, randomised, controlled, parallel group trial in 110 patients with primary Sjögren’s Syndrome exhibiting both fatigue and oral dryness to evaluate the use of two courses of rituximab compared to placebo infusion, in addition to standard therapy. Patients will be followed-up for up to 48 weeks (see Figure 1).

### Recruitment

Participant recruitment is planned over a 36-month period from a maximum of 25 centres across the United Kingdom. Patients will be approached by a research clinician during standard clinic visits. Alternatively patients may be identified by case note review and the UKPSSR and sent a Patient Invitation Letter inviting them to take part. Patients will be provided with verbal and written details about the trial (detailed Patient Information Sheet). Patients will be given at least 24 hours to read the information to consider participation. Patients will be invited to provide written informed consent before being formally assessed for eligibility by the Principal Investigator or any other qualified member of the trial team. Eligible patients from centres that have access to ultrasound and also have the facilities to perform gland biopsies will be invited to take part in the optional sub-studies to obtain minor labial gland biopsies and labial salivary gland ultrasound scans.

### Screening

Patients will be registered for the study before any trial-related invasive or non-invasive procedures are performed. All patients will undergo a screening assessment within 28 days prior to the start of treatment to determine eligibility for the study. Patients need to satisfy all of the study inclusion criteria and not have any of the exclusion criteria in Table 1. Patients who are initially ineligible for the trial may be re-screened at a later date. This applies to patients with low or no salivary flow, have no systemic features (but have disease duration of 10+ years) or have a fatigue and/or oral dryness rated as below 5 on a Likert scale of 0–10.

**Table 1:** Eligibility criteria for randomisation into the TRACTISS trial

**Inclusion criteria**	
1	Aged between 18 and 80 years of age.
2	A confirmed diagnosis of primary Sjögren’s Syndrome by AECG criteria.
3	Positive for anti-Ro auto-antibodies.
4	Patients with a diagnosis of primary Sjögren’s Syndrome (by AECG criteria) with more than 10 years disease duration must have at least one systemic feature of*:*
	• Hypergammaglobulinaemia (IgG over 16)*, or
	• Low complement C4*, or
	• Cryoglobulinaemia
	**OR**
	• Active/past history since diagnosis of the following (ascribed to Sjögren’s Syndrome):
	○ Purpura/cutaneous vasculitis,
	○ Lymphadenopathy,
	○ Persistent parotid salivary gland swelling not due to infection,
	○ Peripheral neuropathy (previously documented by nerve conduction tests),
	○ Interstitial lung disease confirmed by HRCT,
	○ Renal tubular acidosis requiring treatment,
	○ CNS disease ascribed to Sjögren’s syndrome (confirmed by MRI),
	○ Myositis (CPK>2N and EMG or biopsy evidence of myositis),
	○ Inflammatory arthritis
5	An unstimulated salivary flow rate greater than 0 ml in 15 minutes.
6	Symptomatic oral dryness (≥ 5/10 on patient-completed Likert**).
7	Symptomatic fatigue (≥ 5/10 on patient-completed Likert**).
8	Patients on corticosteroids, NSAIDS, antidepressants, DMARDs (e.g. methotrexate, hydroxychloroquine, azathioprine, MMF, leflunomide, ciclosporin etc.), or pilocarpine*** must have been on a stable dose for 4 weeks prior to receiving the first infusion of study medication and expected to remain on this dose throughout the study. If they have stopped any of these drugs they should have been off it for at least 4 weeks prior to receiving study medication.
9	Given their written informed consent to participate in the trial and expected to be able to adhere to the study visit schedule and other protocol requirements.
	*Anti-Ro antibody test, IgG, RF and C4 assays performed within 6 months of screening may be used to confirm eligibility. If greater than 6 months repeats should be performed locally at screening to confirm eligibility.
	**LIKERT range 0-10 with 10 corresponding to worst severity.
	***Pilocarpine or drugs with similar pharmacological action should not be used within 12 hours of the assessment visits at screening, baseline, week 16, week 24, week 36 and week 48 (end of study).
**Exclusion criteria**	
	Patients will be excluded from the trial for the following reasons:
1	Diagnosis of secondary Sjögren’s Syndrome.
2	Pregnancy, lactation or women of child-bearing potential (WCBP) unwilling to use medically approved contraception whilst receiving treatment and for 12 months after treatment has finished.
3	Men whose partners are of child-bearing potential but who are unwilling to use appropriate medically approved contraception whilst receiving treatment and for 12 months after treatment has finished.
4	Patient has active or prior hepatitis B or C, known HIV positivity or known history of tuberculosis.
5	Immunodeficiency or neutropaenia <1.5 × 10^9^/l (unless a diagnosis of benign ethnic neutropaenia has been confirmed in which case the neutrophil count must be > 0.9 × 109/l at screening for Jamaican and Afro-Caribbean populations [16]).
6	Any AECG exclusion criteria not covered elsewhere (graft versus host disease, primary lymphoma excluding PSS, sarcoidosis).
7	Any malignancies that would normally preclude the use of rituximab within the past 5 years, including solid tumours, haematological malignancies and carcinoma in situ (except basal cell or squamous cell carcinoma of the skin that has been excised and cured).
8	Participation in a clinical study involving administration of an investigational drug within the past 4 weeks prior to the first infusion.
9	A history of major surgery within 3 months prior to first infusion or planned surgery during the study.
10	Receipt of live/attenuated vaccine within 4 weeks prior to the first infusion.
11	Previous exposure to rituximab or any other monoclonal antibody within the past 5 years.
12	History of recurring or chronic infections or underlying conditions which may further predispose patients to serious infection.
13	History of moderate to severe congestive heart failure according to the New York Heart Association (NYHA) functional classification system or other uncontrolled heart disease, or who have a clinically significant abnormal ECG at the time of screening.
14	History of receiving human/murine recombinant products or known allergy or anaphylactic reaction to a biologic agent or any component of the active substance or any of its excipients or murine components.
15	Patients with fibromyalgia or a diagnosis of significant depression or anxiety that in the opinion of the clinician would confound the interpretation of the study results.
16	Current or a history of severe, progressive or uncontrolled renal, hepatic, hematologic, gastrointestinal, endocrine, pulmonary, cardiac, neurologic, or cerebral disease (including demyelinating diseases such as multiple sclerosis).
17	Any history of organ transplant (with the exception of a corneal transplant >3 months prior to study entry).
18	Presence of a clinically significant illness or mental disorder within 4 weeks of the start of the trial where the safety of the individual might be at risk by entry into the trial, or where the individual does not have the capacity to consent or where the outcome of the therapy cannot be assessed by virtue of the illness or disorder. Each patient will be assessed individually and no person who wishes to participate will be unreasonably excluded by virtue of the illness or disorder.

### Randomisation

Following confirmation of eligibility, patients will be randomised on a 1:1 basis to receive either rituximab or placebo infusion, in addition to standard therapy. Randomisation will be performed using minimisation incorporating a random element, to ensure treatment groups are well-balanced for: Centre; Age (≥65 or <65); Disease Duration (≥10years or <10years); Consent for minor labial gland biopsy; Consent for ultrasound scan. Both registration and randomisation will be performed centrally using an automated 24-hour telephone system based at the Leeds Clinical Trials Research Unit.

Participating research sites will be required to complete a log of all patients over the age of 18 with PSS who are not registered for screening nor randomised either because they are ineligible or because they decline participation.

### Blinding

The double-blind status of the trial will be maintained by ensuring rituximab and placebo infusion bags are identical in appearance at the time of dispensing from Pharmacy. Treatment allocation will only be known to the trial pharmacy and the central safety team at the CTRU. The Investigators and other members of the site staff involved with the trial will remain blinded to the allocation. All patients will receive the same pre- and post-infusion medication, and the infusion will be administered in the same way for both groups.

Emergency unblinding is permitted where knowledge of treatment allocation is necessary for the appropriate medical management of the patient. This will be performed by the safety team at the CTRU (during office hours) or the local Pharmacy team (during out of office hours). Causality and expectedness of Adverse Events/Serious Adverse Events will be assessed as though the patient received rituximab.

### Treatment schedule

**Figure 2:**
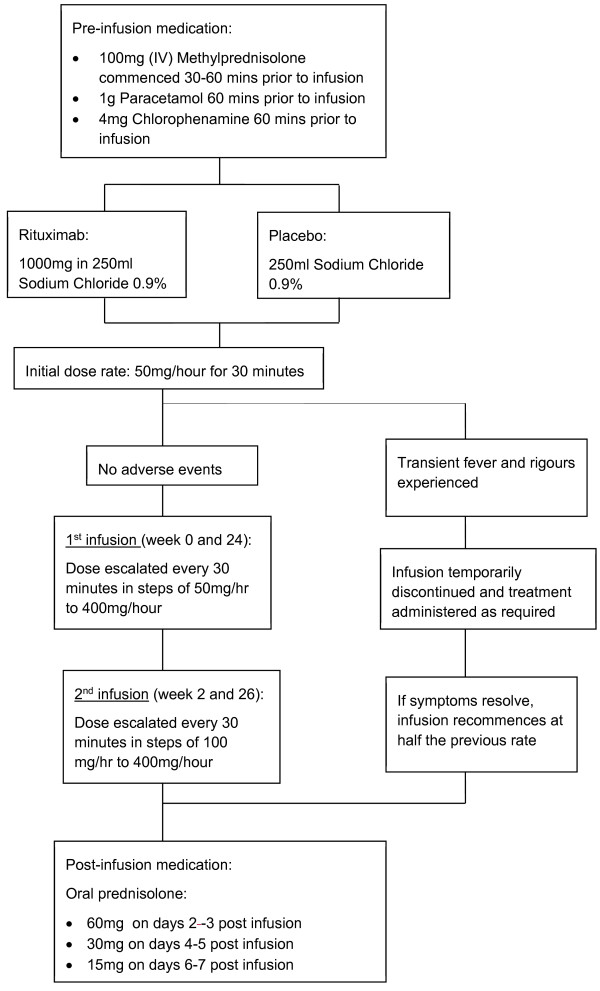
Treatment schedule.

Patients will receive two courses of rituximab or placebo. Each course consists of two doses of rituximab or placebo by intravenous infusion, administered at a two-week interval. The first course is at week 0 and week 2; the second course is at week 24 and week 26. See Figure 2 for details of the treatment schedule.

### Assessments, samples and data collection

All protocol-required assessments will be entered onto paper case report forms at each site. All timings relate to first dosing at Week 0 (Day 1). An assessment of response is made at week 16, week 24, week 36 and week 48 (end of study).

**Figure 3:**
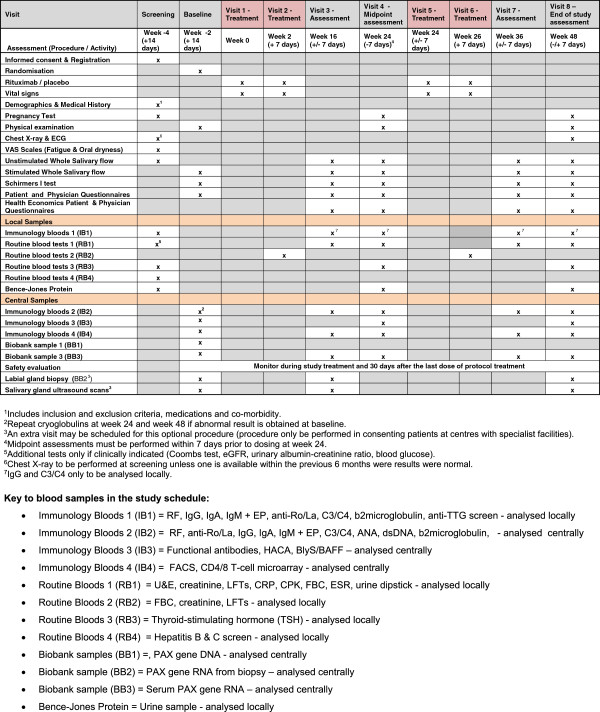
Schedule of events.

The trial visits are structured as detailed in Figure 3.

• Screening visit: All patients will undergo screening within 28 days prior to the start of treatment.

• Baseline visit: Baseline assessments are to be performed after eligibility has been confirmed and within 14 days prior to the start of treatment.

• Treatment visits: All treatment visits will take place at weeks 0, 2, 24 and 26.

• Assessment visits: Patients will be assessed at weeks 16, 24, 36 and 48 for safety and outcome measures and 7 days prior to dosing at weeks 24 for safety.

• Biological samples collected from participants will be sent to the Central Laboratory at the William Harvey Research Institute, London for processing, appropriate analysis and storage. These samples will be used for a range of studies of direct relevance to the treatment of PSS.

### Sample size

A total of 110 patients will be required for this study. A sample size of 50 evaluable patients per treatment group will provide 80% power to detect a difference in response rates of 30% corresponding to 25% on placebo and 55% on rituximab, assuming a 2-sided 5% significance level. 50 patients per group will also provide at least 80% power to detect a between group difference of 30% if the placebo group event rate is as high as 50% (50% placebo and 80% rituximab). In order to allow for a 10% attrition rate, 110 patients will be randomised.

### Outcomes

The primary outcome is achievement of a 30% reduction at 48 weeks from baseline in either oral dryness or fatigue measured using visual analogue scales (VAS - Range 0-100 mm).

Secondary outcomes at weeks 16, 24, 36 and 48 are: the values of the fatigue and oral dryness scales (VAS), ocular dryness (VAS), patient global assessment of health (VAS), physician global assessments of disease activity and disease damage (0–10 Likert scales), stimulated and unstimulated salivary flow rates, lacrimal flow (Schirmers I test of ocular function), and the EULAR Sjögren’s Syndrome Patient Reported Index (ESSPRI). In addition, further quality of life, and disease damage and disease activity indices at weeks 24 and 48 will be: Medical Outcomes Survey Short Form (SF-36), EQ-5D, EULAR Sjögren’s Syndrome Disease Activity Index (ESSDAI), and the Profile of Fatigue and Discomfort – Sicca Symptoms Inventory (PROFAD-SSI). Outcomes relating to systemic disease activity features at weeks 24 and 48 are: Sjögren’s Syndrome Clinical Activity Index (SCAI), Sjögren’s Syndrome Disease Activity Index (SSDAI), Sjögren’s Syndrome Disease Damage Index (SSDDI), and Sjögren’s Syndrome Damage Index (SSDI). Further outcomes correspond to serological and peripheral blood biomarkers (haematology, biochemistry, serology and immunology assays) measured at each visit. Both patients and physicians complete an exit poll at week 48 asking them if they have suspicions as to which treatment the patient received. Outcomes relating to the optional sub-studies (Glandular histology and composition assessed by biopsy and ultrasound respectively) will be measured at weeks 16 and 48, and the T-Cell sub-study outcomes (peripheral and lesional T cells) will be assessed at week 16 for analysis in this study with samples taken at weeks 24, 36 and 48 for use in future studies.

### Statistical analysis

All analyses will be conducted on the intention-to-treat (ITT) population and patients will be analysed according to the treatment group to which they were randomised. All formal analyses will be carried out at a 2-sided 5% level of significance.

#### Primary outcome analyses

A binary logistic regression approach will model the odds of achieving a 30% reduction in symptoms for patients receiving rituximab compared to placebo. Models will include baseline scores for oral dryness and fatigue, as well as disease duration (≤10 years/>10 years), age and centre. The estimated odds ratio for treatment effect will be presented, with the associated 95% confidence interval, and p-value from a likelihood ratio test comparing the models with and without the treatment variable. In addition, the number and proportion of patients in both the rituximab and placebo groups who achieve a reduction in either oral dryness or fatigue >30% between the end of study visit (week 48) and the baseline visit will be summarised. Multiple imputation will be used to impute missing primary endpoint data.

#### Secondary outcome analyses

• Fatigue, oral and ocular dryness, patient and physician global assessments: For each symptom, a multi-level repeated measures linear regression model will be used to compare VAS scores between groups, models will include the minimisation factors (age, centre, disease duration).

• Salivary and Lacrimal Flow: The flow rates (stimulated salivary flow, unstimulated salivary flow, lacrimal flow) will be compared between the treatment groups using a multi-level repeated measures linear regression model, models will include the values at baseline and the minimisation factors.

• Quality of Life and Disease Activity Indices: The change in each Quality of Life Score, from baseline to 24 and 48 weeks, as measured by SF-36 and each Disease Activity Index (ESSDAI, ESSPRI, SF-36 and PROFAD-SSI) will be compared between treatment groups using a linear regression model, including baseline score values and the minimisation factors.

• Safety and toxicity: Safety analyses will summarise the adverse events (including serious adverse events (SAEs), serious suspected adverse reactions and suspected unexpected serious adverse events), laboratory changes and treatment-related mortality rates. Safety data will be presented by treatment group and relationship to study treatment or underlying PSS. Expected SAEs related to rituximab include infusion-related reactions, infections, cardiovascular events, hepatitis B reactivation and Progressive Multifocal Leukoencephalopathy.

#### Exploratory analyses

Systemic disease activity features: Recently developed measures (SCAI, SSDAI, SSDDI and SSDI) will be summarised for each time-point at which they were measured.

#### Economic evaluation

The primary objective of the economic evaluation is to identify the within study incremental cost effectiveness ratios; the difference in costs between rituximab and placebo divided by the difference in benefits between rituximab and placebo. Analysis will use quality adjusted life years (QALYs) as the summary measurement of benefits. The estimation of QALYs requires the production of utility weights for each health state observed in the trial population. The EQ-5D (Euroqol) instrument will be used for this purpose and estimation of QALYs will use the area under the curve method [17,18]. Secondary analysis will use the SF-36. Resource utilisation will be captured at 16, 24, 36 and 48 weeks after randomisation.

Sensitivity analysis of the incremental cost effectiveness ratio will be undertaken. Bootstrapped incremental costs and benefits will be calculated and used to estimate their joint distribution. These estimates will form a scatterplot on the cost effectiveness plane, and will be used to estimate a 95% cost effectiveness ellipse for the joint distribution of increments costs and benefits, as well as the cost effectiveness acceptability curve for a range of values that a service provider may be willing to pay for one additional QALY. Given the duration of the study discounting is not required.

## Discussion

PSS is characterised by oral and ocular dryness, severe fatigue with reduced quality of life, B-cell hyperactivity, anti-Ro/La antibodies and a 44 times increased risk of B-cell lymphoma. There is no effective systemic therapy that can alter the progress of the disease and no immediate prospect of commercial studies.

This protocol describes a phase III study of 110 patients treated with either rituximab or placebo with re-treatment at 6 months. Although small for a phase III trial it has realistic recruitment and outcome objectives given the prevalence of Sjögren’s syndrome, and it will be the largest trial conducted in the UK to date. Furthermore, the broadly similar patient population and design of the French study by Saraux *et al*. [13] should allow for future meta-analysis of the data.

In parallel with the clinical outcomes of improvement in fatigue and/or dryness the TRACTISS study includes a number of biological outcomes including glandular immunohistochemistry and genetic features, alteration in anti-muscarinic antibody levels, peripheral T-cell numbers and transcriptional profile, and BAFF/BLyS levels. The availability of anti-B-cell therapy for RA means we can also study whether it works in treating the debilitating symptoms of PSS and this is the goal of this study. In addition we will maximise the benefit of the study by also measuring changes in the glands and blood of patients through additional scientific research work that will add support to the clinical findings.

If the results of this study are positive this could lead to anti-B-cell therapy becoming part of routine clinical care for patients with PSS and/or opening new avenues for developing alternative disease-modifying therapeutic approaches for a disease that currently has none available. The incorporated integral scientific components of TRACTISS will maximize the added value of the study to better understand the pathological processes by looking at the effects of rituximab on the glands, blood cells and antibodies in PSS and guide future therapeutic research.

### Trial status

The first patient was enrolled into TRACTISS on the 25th August 2011 and recruitment is due to end December 2013. The study is being conducted in 25 sites across the UK. We expect to report the main trial results in Autumn 2015. Ethical and governance approval for this trial has been obtained from the Leeds West Ethics Committee (ref 10/H1307/99) and the Leeds Teaching Hospitals NHS Trust respectively. The trial progress is monitored by an independent Data Monitoring and Ethics Committee (DMEC) and Trial Steering Committee (TSC).

## Abbreviations

AECG: American-European Consensus Group; Anti-TNF: Anti-tumor necrosis factor; BAFF: B-cell activating factor; BLyS: B Lymphocyte stimulator; CLL: Chronic lymphocytic leukaemia; CTRU: Clinical trials research unit; DMEC: Data monitoring and ethics committee; EQ-5D: European quality of life questionnaire (Five Domain Version); ESSDAI: EULAR Sjögren’s syndrome disease activity index; ESSPRI: EULAR Sjögren’s syndrome patient reported index; EULAR: European league against rheumatism; ITT: Intention to treat; IV: Intra ventricular; LIHS: Leeds institute of health sciences; MTX: Methotrexate; NHL: Non-Hodgkin’s leukaemia; PROFAD-SSI: Profile of fatigue and discomfort – Sicca symptoms inventory; PSS: Primary Sjögren’s syndrome; QALYs: Quality adjusted life years; RA: Rheumatoid arthritis; SAE: Serious adverse event; SCAI: Sjögren’s syndrome clinical activity index; SF-36: Medical outcomes survey short form; SLE: Systemic lupus erythematosus; SSDAI: Sjögren’s syndrome disease activity index; SSDDI: Sjögren’s syndrome damage index; SSDDI: Sjögren’s syndrome disease damage index; TSC: Trial steering committee; UKPSSR: UK primary Sjogren’s syndrome registry; VAS: Visual analogue scale.

## Competing interests

The authors declare that they have no competing interests.

## Authors’ contributions

SBo, CPi and PE designed the trial with input from the trial team. WN contributed to the study design. NNC led the drafting of the protocol and was responsible for study set up and management. ES was responsible for the overall management of the study during the protocol development stage. SP participated in the overall design of the study and helped to draft the protocol. JG participated in the design of the study protocol with responsibility for the design of the study protocol. MB, RB and FH participated in the design of the study protocol, with particular responsibility for the subgroup, labial gland biopsy and T-cell sub-study analyses. JR and IM participated in the design of the study protocol, with particular responsibility for the design of the ultrasound component of the protocol. APG and SR participated in the design of the oral and ocular components of the protocol. LD, PE, CPi, CPe, EP, PS, and NS participated in the coordination of the design of the protocol. SBr is responsible for providing statistical supervision for the study. CE has produced the statistical analysis plan and is responsible for conducting the statistical analysis and reporting of the study. CH participated in the design of the study protocol, with particular responsibility for the health economics. SR was responsible for the study set-up and study coordination. CR is primarily responsible for the acquisition of clinical data. CW is the PPI representative and contributed to the design of the study. NNC produced a first draft of the protocol paper and SBr produced subsequent drafts and coordinated the review for publication. All members of the TRACTISS study team had the opportunity to comment and approved the final manuscript.

## Pre-publication history

The pre-publication history for this paper can be accessed here:

http://www.biomedcentral.com/1471-2474/15/21/prepub
